# Polymorphisms of genes coding for ghrelin and its receptor in relation to colorectal cancer risk: a two-step gene-wide case-control study

**DOI:** 10.1186/1471-230X-10-112

**Published:** 2010-09-28

**Authors:** Daniele Campa, Barbara Pardini, Alessio Naccarati, Ludmila Vodickova, Jan Novotny, Verena Steinke, Nils Rahner, Elke Holinski-Feder, Monika Morak, Hans K Schackert, Heike Görgens, Judith Kötting, Beate Betz, Matthias Kloor, Christoph Engel, Reinhard Büttner, Peter Propping, Asta Försti, Kari Hemminki, Roberto Barale, Pavel Vodicka, Federico Canzian

**Affiliations:** 1 Genomic Epidemiology Group, German Cancer Research Center (DKFZ), Im Neuenheimer Feld 280, D-69120 Heidelberg, Germany; 2 Department of Biology, University of Pisa, Pisa, Italy; 3 Institute of Experimental Medicine, Academy of Sciences of the Czech Republic, Prague, Czech Republic; 4 Department of Oncology, First Faculty of Medicine, Charles University, Prague, Czech Republic; 5 Institute of Human Genetics, University of Bonn, Bonn, Germany; 6 Department of Internal Medicine, Campus Innenstadt, University Hospital of Ludwig-Maximilians-University, Munich, Germany; 7 Department of Surgical Research, Technische Universität Dresden, Dresden, Germany; 8 Medical Department, Knappschaftskrankenhaus, Ruhr University, Bochum, Germany; 9 Institute of Human Genetics, University of Düsseldorf, Düsseldorf, Germany; 10 Department of Applied Tumour Biology, Institute of Pathology, University of Heidelberg, Heidelberg, Germany; 11 Institute of Medical Informatics, Statistics and Epidemiology, University of Leipzig, Leipzig, Germany; 12 Institute of Pathology, University of Bonn, Bonn, Germany; 13 Center for Primary Health Care Research, Clinical Research Center, Lund University, Malmö, Sweden

## Abstract

**Background:**

Ghrelin, an endogenous ligand for the growth hormone secretagogue receptor (GHSR), has two major functions: the stimulation of the growth hormone production and the stimulation of food intake. Accumulating evidence also indicates a role of ghrelin in cancer development.

**Methods:**

We conducted a case-control study to examine the association of common genetic variants in the genes coding for ghrelin (GHRL) and its receptor (GHSR) with colorectal cancer risk. Pairwise tagging was used to select the 11 polymorphisms included in the study. The selected polymorphisms were genotyped in 680 cases and 593 controls from the Czech Republic.

**Results:**

We found two SNPs associated with lower risk of colorectal cancer, namely SNPs rs27647 and rs35683. We replicated the two hits, in additional 569 cases and 726 controls from Germany.

**Conclusion:**

A joint analysis of the two populations indicated that the T allele of rs27647 SNP exerted a protective borderline effect (P_trend _= 0.004).

## Background

Ghrelin, an endogenous ligand for the growth hormone secretagogue receptor (GHSR), is a 28-amino residue peptide predominantly produced by the stomach [1]. In addition to the mature form of ghrelin, several post-transcriptional and post-translational variants have been reported [2]. Two molecular forms (ghrelin and des-acyl ghrelin) resulting from a different post-transcriptional modification of the protein, are observed in human plasma. The ghrelin receptor has two known isoforms: one which is functional (GHSR-1a) and one spliced variant (GHSR-1b) with no known function [3]. Only the acylated form of ghrelin can bind the GHSR-1a receptor [1]. Two main functions of ghrelin are documented: first to stimulate growth hormone (GH) production through the activation of the GHSR-1a in the hypothalamus [4] and second to increase appetite and food intake [5,6] by mechanisms that could be independent of GHSR [7].

Circulating ghrelin levels are correlated with obesity, and insulin may be an important regulator of plasma ghrelin levels in different states of nutrition [8-11]. Several studies of different populations have shown that levels of ghrelin are related to body size [12], although its mode of action as a regulator of body fat stores remains unclear [12]. Obesity induces a number of metabolic disturbances known as the metabolic syndrome, and is associated with an excess risk of insulin resistance, diabetes, and cardiovascular disease [13-15].

Obesity and related metabolic abnormalities are consistent risk factors for CRC [16]. In most studies, obesity (measured as BMI, waist circumference or waist-to-hip ratio) is associated with a relative risk of 1.5 to 2.0 compared with a low or normal BMI [17-20]. Similarly, associations for circumference measures have been noted for large or advanced adenoma, the proximate precursor to most colon cancers [19,21,22]. Overall, the data strongly support that some metabolic characteristics associated with central or abdominal adiposity increases risk of CRC.

Accumulating evidence also indicate a role of ghrelin in cell proliferation, inhibition of apoptosis and cancer development [23-26].

Polymorphisms in the coding region of the ghrelin gene were suggested to be involved in the aetiology of obesity and to modulate glucose-induced insulin secretion in different ethnic study groups [27]. Hence, variations in the ghrelin gene influencing the expression and/or function of the ghrelin protein might alter energy balance, contribute to obesity and, indirectly, to CRC risk. We postulate that Single Nucleotide Polymorphisms (SNPs) in the genes coding for ghrelin and its receptor maybe associated with an altered risk of CRC.

In this report we investigated the genetic variability of the *GHRL *and *GHSR *genes. Using a tagging approach and selecting 7 SNPs in *GHRL *gene and 4 SNPs in the *GHSR *gene we covered all the known genetic variation of the two genes. We tested the impact of *GHRL *and *GHSR *SNPs on CRC risk in a case-control study based on subjects from the Czech Republic. In a second step we replicated the best associations in an unrelated German population. To our knowledge this is the first report on polymorphisms of *GHRL*, *GHSR *and CRC risk.

## Methods

### Study populations

In this study we have used two distinct populations: one from Czech Republic and the other from Germany. All SNPs were typed in the Czech population while only two SNPs showing an altered risk of CRC in the first population were typed in the German cases and controls.

### Czech population

The population has been extensively described elsewhere [28,29]. Briefly: cases were CRC patients visiting nine oncological departments (two in Prague, one each in Benesov, Brno, Liberec, Ples, Pribram, Usti nad Labem, and Zlin) distributed in all geographic regions of Czech Republic and being representative of the population of the entire country. This study includes 680 patients who could be interviewed and provided biological samples of sufficient quality for genetic analysis. All cases had histological confirmation of their tumor diagnosis.

Controls were selected among patients admitted to five large gastroenterological departments (Prague, Brno, Jihlava, Liberec, and Pribram) all over the Czech Republic, during the same period of the recruitment of cases. Selected controls were all of Czech Caucasian origin. Only subjects whose colonoscopic results were negative for malignancy, colorectal adenomas or IBD were chosen as controls. Among 739 invited controls, a total of 593 (80.2%) were analyzed in this study (lost controls were similar to those included with respect to sex distribution).

Cases included in this study had a mean age of 61 years (range 27-90), while controls had a mean age of 56 years (range 28-91). Study subjects provided information on their lifestyle habits (smoking, drinking, diet etc.), and family/personal history of cancer, with the use of structured questionnaires.

The genetic analyses did not interfere with diagnostic or therapeutic procedures for the subjects. All participants signed an informed written consent and the design of the study was approved by the Ethical Committee of the Institute of Experimental Medicine, Prague, Czech Republic.

### German population

CRC cases comprised 569 German Caucasian index patients (age range 9-88 years, mean 43.6 years) recruited by the six German university hospitals of Bochum (BO), Bonn (BN), Dresden (DD), Düsseldorf (DÜ), Heidelberg (HD) and Munich/Regensburg (MR). Cases were collected as part of a large study on susceptibility to hereditary nonpolyposis CRC (HNPCC). Inclusion criteria for the cases were (i) a family history of CRC or (ii) CRC diagnosed under the age of 50. Analysis for microsatellite instability was applied as a pre-screening test prior to mutation analysis in the MSH2 and MLH1 genes. All cases were tested to be microsatellite stable.

The control series consisted of 726 healthy, unrelated, sex-and age matched blood donors (26-68 years, mean 45.9 years) which were recruited between 2004 and 2006 by the Institute of Transfusion Medicine and Immunology, Faculty of Mannheim, Germany. The matching intervals for age were 'younger than 30 years', five-year groups (30-34, 35-39, ..., 60-64) and 'older than 65 years'. Blood sampling was performed during regular blood donation according to German guidelines. Selected controls were all of German Caucasian origin. The study was approved by the competent local Ethics Committees, and written informed consent was obtained from all individuals.

### Selection of tagging SNPs

We aimed at surveying the entire set of common genetic variants in the *GHRL *and *GHSR *genes. For this purpose, we used the Tagger algorithm [30] that was developed to select maximally informative sets of tagSNPs in candidate-gene association study. All polymorphisms in the region of the two genes of interest with minor allele frequency (MAF) ≥5% in Caucasians from the International HapMap Project (version 22; http://www.hapmap.org), were included. Tagging SNPs were selected with the use of the Tagger program within Haploview http://www.broad.mit.edu/mpg/haploview/; http://www.broad.mit.edu/mpg/tagger/[31,32], using pairwise tagging with a minimum r^2 ^of 0.8.

This resulted in a selection of 11 tagging SNPs, 7 for the *GHRL *gene (with a mean r^2 ^of the selected SNPs with the SNPs they tag of 0.967), and 4 for the *GHSR *gene (with an r^2 ^of 0.989). Our selection thus captures to a very high degree the known common variability in this gene.

### DNA extraction and genotyping

DNA was extracted from blood samples with standard proteinase K digestion followed by phenol/chloroform extraction and ethanol precipitation. The order of DNAs from cases and controls was randomized on PCR plates in order to ensure that an equal number of cases and controls could be analyzed simultaneously. All the genotyping was carried out using the Taqman assay, according to manufacturer's protocol. The pre-designed Taqman assays were purchased from Applied Biosystems (Foster City, CA).

All samples that did not give a reliable result in the first round of genotyping were resubmitted to up to two additional rounds of genotyping. Data points that were still not filled after this procedure were left blank. Repeated quality control genotypes (8% of the total) showed an average concordance of 99.5%.

### Statistical Analysis

The frequency distribution of genotypes was examined for the cases and the controls. Hardy-Weinberg equilibrium was tested in the cases and in the controls separately by chi square test. We used logistic regression for multivariate analyses to assess the main effects of the genetic polymorphism on CRC risk using a codominant inheritance model. The most common allele in the controls was assigned as the reference category. All analyses were adjusted for age and sex.

Additionally, we performed a logistic regression stratifying for the cancer site (colon *versus *rectum) and smoking (smokers *versus *non smokers and heavy smokers *versus *light smokers) or alcohol drinking (drinkers *vs*. non-drinkers) habits.

For SNPs rs27647 and rs35683 the analysis were performed in the two populations. Odds ratios were calculated for the two populations separately and jointly.

All the analyses were performed with STATA software (StataCorp, College Station, TX).

## Results

We performed a case-control study using two different sets of SNPs in two distinct populations of German and Czech origins. The first SNP set was made of 11 tagging SNPs which we tested in 680 cases and 593 controls from the Czech Republic. The second SNP set consisted of the two best hits, namely SNPs rs27647 and rs35683, which we replicated in additional 569 cases and 726 controls from Germany. The genotype frequencies among the controls were in Hardy-Weinberg equilibrium for all the SNPs and in both populations.

### Results for the Czech population

The distribution of the genotypes and their odds ratios (ORs) for association with CRC risk are shown in Table 1.

**Table 1:** Associations of *GHRL *and *GHSR *polymorphisms with colorectal cancer risk in the Czech population

	Cases^a^	Controls^a^	OR(95%)^b^	P value	P trend
***GHRL***					
					
rs26802					
G/G	286	278	1		
G/T	294	245	1.18(0.92-1.51)	0.18	
T/T	93	74	1.19(0.82-1.70)	0.25	0.16
rs27647					
C/C	279	198	1		
C/T	289	280	0.70(0.55-0.91)	0.008	
T/T	93	110	**0.57(0.40-0.80)**	0.002	**0.001**
rs35683					
A/A	204	147	1		
A/C	331	297	0.80(0.61-1.05)	0.11	
C/C	137	143	**0.71(0.51-0.98)**	**0.04**	**0.02**
rs4684677					
A/A	601	523	1		
A/C	78	74	0.91(0.64-1.30)	0.49	
C/C	4	3	1.63(0.35-7.68)	0.62	0.70
rs696217					
G/G	590	523	1		
G/T	84	71	1.09(0.77-1.55)	0.64	
T/T	4	6	0.60(0.16-2.18)	0.43	0.89
rs2075356					
C/C	559	498	1		
C/T	101	78	1.20(0.86-1.68)	0.63	
T/T	7	7	0.88(0.30-2.61)	0.36	0.28
rs35684					
A/A	257	248	1		
A/G	213	174	1.10(0.84-1.43)	0.46	
G/G	47	34	1.24(0.77-2.00)	0.36	0.29

***GHSR***					
					
rs2922126					
T/T	255	20	1		
A/T	269	274	0.81(0.62-1.05)	0.11	
A/A	74	39	1.48(0.95-2.30)	0.82	0.31
rs2944694					
A/A	564	477			
A/G	98	101	0.78(0.57-1.07)	0.14	
G/G	7	11	0.46(0.16-1.28	0.14	0.08
rs495225					
A/A	333	293	1		
A/G	232	220	0.88 (0.68-1.13)	0.33	
G/G	58	42	1.17(0.75-1.83)	0.47	0.77
rs572169					
C/C	329	305	1		
C/T	263	235	1.03(0.80-1.31)	0.80	
T/T	57	48	1.14(0.74-1.74)	0.55	0.62

^a ^Numbers may not add up to 100% of subjects due to genotyping failure. All samples that did not give a reliable result in the first round of genotyping were resubmitted to up to two additional rounds of genotyping. Data points that were still not filled after this procedure were left blank.

^b ^OR: odds ratio; CI: confidence interval. Adjusted for age and gender. Results in bold are statistically significant (p < 0.05)

We found that, in this sample set, carriers of the T allele of SNP rs27647 had a decreased risk of CRC, with an OR of 0.70 (95% confidence interval (95% CI) 0.55-0.91; P_value _= 0.013), for C/T heterozygous individuals and an OR of 0.57 (95% CI 0.40-0.80; P_value _= 0.002) for T/T homozygous individuals (P_trend _= 0.001).

Moreover we found that carriers of the C allele of SNP rs35683 had a decreased risk of CRC, with an OR of0.71 (95% CI 0.51-0.98; P_value _= 0.04) for C/C homozygous individuals. The OR of 0.80 for the heterozygous individuals was not statistically significant (95% CI 0.60-1.05; P_value _= 0.42) (P_trend _= 0.02).

We did not find any statistically significant association between the other SNPs and CRC risk.

### Results for the German population

The distribution of the genotypes and their odds ratios (ORs) for association with CRC risk are shown in Table 2.

**Table 2:** Associations of *GHRL *polymorphisms with colorectal cancer risk in the German cases and controls.

	Cases^a^	Controls^a^	OR(95%)^b^	P value	P trend
rs27647 ***GHRL***					
C/C	213	259	1		
C/T	278	360	0.93(0.73-1.19)	0.60	
T/T	78	107	0.83(0.62-1.24)	0.49	0.54

rs35683 ***GHRL***					
A/A	172	220	1		
A/C	277	356	0.99(0.77-1.28)	0.97	
C/C	120	150	0.99(0.72-1.35)	0.95	0.99

^a ^Numbers may not add up to 100% of subjects due to genotyping failure. All samples that did not give a reliable result in the first round of genotyping were resubmitted to up to two additional rounds of genotyping. Data points that were still not filled after this procedure were left blank.

^b ^OR: odds ratio; CI: confidence interval. Adjusted for age and gender. Results in bold are statistically significant (p < 0.05)

The associations found in the Czechs were not confirmed in the German population. However, the effect of SNP rs27647 was statistically significant in a joint analysis of data from the two populations. The carriers of the T allele in the joint group exerted a protective effect, with an OR of 0.82 (95% CI 0.69-0.98; P_value _= 0.02), for C/T individuals and an OR of 0.73 (95% CI 0.58-0.93; P_value _= 0.01) for T/T homozygous individuals (P_trend _= 0.0043).

Applying the Bonferroni correction for multiple testing the P_trend _of rs27647 in the joint population remained borderline significant (P_trend _= 0.0043 × 11 = 0.047), although neither the ORs for heterozygotes nor for the homozygotes remained statistically significant after correction. A test for heterogeneity indicated that the results for the two populations were statistically different (P_heterogeneity _= 0.014).

### Stratified analysis and interactions

Analyses stratified by cancer site (colon vs. rectum), alcohol and smoking habits did not show any significant interaction with polymorphisms (data not shown).

We performed analysis stratified by age using the median age as a cut off. In the German population we found no difference in the genotype distribution in the two population strata concerning cancer risk. For the Czech population we found that only in the older group (age >66) the homozygous carriers of the variant alleles of two SNPs showed a statistically significant association with cancer risk: OR of 0.39 (95% CI 0.21-0.73 P_value _= 0.003) for SNP rs27647 and OR of 0.46 (95% CI 0.28-0.78 P_value _= 0.004) for SNP rs35683.

Finally we performed an analysis combining the two polymorphisms in order to assess the impact of cancer risk of a multi-locus risk score, but the results did not explain more of the genetic susceptibility to the disease than the two SNPs alone (data not shown).

## Discussion

Recent evidence indicates that obesity and related metabolic abnormalities are associated with increased incidence and mortality for CRC. Since it has been shown that circulating levels of ghrelin are related to body size we postulated that polymorphisms that could alter protein expression and/or function may also alter CRC risk.

In this study we investigated the genetic variability of the *GHRL *and *GHRS *genes in relation to CRC risk using a tagging approach and selecting 11 SNPs. Using this method we covered all the known genetic variation of the genes, an effort lacking from all previous studies.

On the first phase of the project we typed all the 11 tagging SNPs in CRC cases and controls from the Czech Republic and we found that two SNPs (rs27647, rs35683) were associated with a decreased risk of CRC.

SNP rs27647 is situated in the promoter region of the gene and has been found associated with insulin level and obesity [33], while SNP rs35683 is situated in the first intron of the gene and has been found associated with BMI in a Caucasian population[34]. Since BMI, obesity and insulin level are well known risk factors for CRC, the two SNPs may indirectly affect cancer risk. We sought to replicate these findings in an independent group and we used a German population with a similar sample size. In this second population the findings were not replicated.

There are two possible explanations for these findings: either the associations observed from the Czech population are false positives, or the differences in the association results are due to differences between the two selected populations. We can confidently exclude a major role of ethnic differences. According to Globocan [35-37], the Czech and the German populations have a comparable CRC incidence. Moreover in a recent study Nelis and colleagues investigated the underlying population stratification in Europe showing that there were very little, if any, differences in the genetic make-up of Germans and Czechs [38]. Another explanation for the inconsistent findings may be due to different environmental factors in the two countries. However dietary habits and food intake are not dramatically different in the two countries http://faostat.fao.org/site/609/DesktopDefault.aspx?PageID = 609. It has to be noted that the German subjects were on average young and had a family history of CRC, whereas the Czech cases were unselected.. It may be speculated that genetic predisposition to familial and sporadic cases is due to groups of genetic variants that do not overlap entirely. According to this hypothesis, rs27647 could be more relevant for sporadic cases but not for familial ones. In addition if ghrelin acts indirectly through the effects of increased BMI, Germans subjects may not have been old enough to have had sufficient exposure to increased BMI to show the increased incidence of cancer. In fact, when we stratified the analysis for age groups, only in the older patients the observed association remained significant. Finally, it has not to be overlooked that ORs for rs27647 were similar in the two groups, but the effect does not reach statistical significance in the Germans,. This may indicate that the association could be true, and a larger, independent study is needed to confirm or disproof this finding.

## Conclusion

In conclusion, we are not able to completely exclude a possible effect of the rs27647 SNP in CRC risk, while we can confidently exclude a major role for the other common SNPs in *GHRL *and *GHSR *as CRC risk factors.

## Competing interests

The authors declare that they have no competing interests.

## Authors' contributions

DC conceived the study, carried out the genotyping the statistical analysis and drafted the manuscript, FC supervised the genotyping and the SNPs selection, VS, NR, EH-F, MM, HS HG, JK, BB, MK, CE, RB, PP, PV, BP, AN, MC, LV, JN enrolled the subjects of the study and helped in the manuscript writing, KH, RB, AF helped in writing the manuscript. All authors read and approved the manuscript.

## Pre-publication history

The pre-publication history for this paper can be accessed here:

http://www.biomedcentral.com/1471-230X/10/112/prepub
